# Transcriptome sequencing of *Cocos nucifera* leaves in response to *Rhynchophorus ferrugineus* infestation

**DOI:** 10.3389/fgene.2023.1115392

**Published:** 2023-02-07

**Authors:** Li Liu, Wei Yan, Bo Liu

**Affiliations:** Hainan Key Laboratory of Tropical Oil Crops Biology/Coconut Research Institute of Chinese Academy of Tropical Agricultural Sciences, Wenchang, China

**Keywords:** red palm weevil, ROS scavenging, herbivore, coconut, host-plant resistance, flavonoid biosynthesis

## Abstract

Red palm weevil (RPW, *Rhynchophorus ferrugineus*) is an invasive pest of palms. In China, coconut (*Cocos* nucifera) production is being significantly affected by the RPW attack. To develop a long-term RPW control strategy, host-plant resistance is the most sustainable option. In this regard, the availability of transcriptome sequencing data from RPW-infected coconut plants can be highly useful. Therefore, the present study assessed coconut leaf physiological responses and transcriptional changes after different days of RPW attack i.e., 5, 10, 15, 20, and 25 days after infestation (DAI). A comparison of physiological data indicated that populations with the higher number of RPW insects i.e., population C (15 males +21 females) and D (20 males +28 females) triggered higher antioxidant enzyme activities. We used this data to study the transcriptomic responses on 5 and 20 DAI. Of the 38,432 detected transcripts, 3,984, 1,981, 3,925, and 2,257 were differentially expressed in CK (control/no RPW)_vs._C (5 DAI), CK_vs._D (5 DAI), CK_vs._C (20 DAI), and CK_vs._D (20 DAI), respectively. These transcripts were enriched in plant-pathogen interaction, phenylpropanoid/flavonoid biosynthesis, amino sugar and nucleotide sugar metabolism, plant hormone signal transduction, mitogen-activated protein kinase, and reactive oxygen scavenging pathway. We discuss these results and present several candidate genes to be manipulated for developing a sustainable strategy to control RPW attack regarding host-plant resistance. Furthermore, these findings provide a basis for developing effective early and late RPW attack detection strategies.

## 1 Introduction

Red palm weevil (RPW, *Rhynchophorus ferrugineus*) is a quarantine pest of palms (Arecaceae). It can spread up to 50 km per day and currently 85 countries and regions have been infested by this insect pest. In China, it was recorded in 1997 in Zhongshan City, Guangdong Province, and has now spread to 15 provinces (Ge et al., 2015). Since its invasion in China, it has damaged >10,000 km^2^ area and killed >20,000 coconut (*Cocos nucifera*) trees. However, the damaged area is rapidly expanding, thus it is not only a danger to the coconut industry but also poses a threat to the ecological security of the Chinese coastal areas. Particularly, the expansion of coconut and *A. catechu* in Hainan province has brought economic benefits to the farmers. Coconut is cultivated on > 40,000 ha in China, of which 99% is located in Hainan province (∼232 million coconuts) (Hainan coconut industry highly quality development: The 14th Five-Year Plan, http://lyj.hainan.gov.cn, accessed on 20.11.2022). However, such increased plantation has also contributed to the rapid spread of RPW (Peng and Hou, 2017). Additionally, the RPW has been included in the “People’s Republic of China Entry Phytosanitary Pest List” (Ministry of Agriculture of the People’s Republic of China, General Administration of Quality Supervision Inspection Quarantine of the People’s Republic of China, 2007) and “National Forestry Quarantine Pest List (Yanhua, 2013)”, and the comprehensive risk evaluation value is highly dangerous.

Due to its habit of dwelling inside the trunks, its early detection is difficult and its presence is confirmed only after the damage symptoms have become visible (Salama et al., 2009). The prevention and control strategies include understanding its population dynamics, chemical control, bait trapping, biological control, and breeding RPW-tolerant genotypes (Peng and Hou, 2017). However, the success of the prevention as well as control of RPW is directly associated with effective early detection (Faleiro, 2006). To detect RPW, computer-assisted tomography, evaluation of the symptoms of larval mines and chewed material at the leaf basis, and bioacoustics sensors have been tested in this regard. However, the feasibility and cost limit the adaptation of these strategies (Giovino et al., 2015). Nevertheless, host-plant resistance is an ideal long-term strategy. Different palm species have been reported to have varying levels of resistance to RPW. Particularly, the reports that show the presence of palm species and wild palms resistant to RPW in Spain and Iran are interesting since they can help us identify the molecular mechanisms of RPW resistance (Barranco et al., 2000; Farazmand, 2002). Additionally, the exploration of host-plant responses to RPW attack on a molecular scale i.e., metabolomic and transcriptomic responses, can help us identify key genes and pathways that can be manipulated for breeding RPW-resistant plants. Studies on *Phoenix canariensis* Chabaud (Giovino et al., 2015), have shown the involvement of genes in phenylpropanoid biosynthesis, fatty acid metabolism, tryptophan metabolism, and hormonal crosstalk. However, such information in coconut cultivars grown in Hainan, China is sparse, thus not allowing a basic understanding of the coconut plants’ response to RPW attack, especially during early infestation stages.

During the co-evolution of plants and herbivore insects, both have evolved certain mechanisms with giving them better survival chances. In the case of plants, when herbivore insects attack, the concentrations of Ca^2+^ change across the membranes (Parmagnani and Maffei, 2022), which initiate a cascade of signaling (Wu and Baldwin, 2010). These signals activate key pathways that produce chemicals to repel herbivores through direct toxicity or affecting the digestibility of plant tissues. Secondly, biosynthesis of several substances is induced as a result of plant tissues’ damage by herbivore insects. These compounds include plant growth regulators e.g., phytohormones, while others include those involved in transport, storage, and as well as primary metabolism related compounds. The signaling pathways involved in herbivory/wounding include plant-hormone signaling and related pathways e.g., MAPK signaling-plant pathway (Mello and Silva-Filho, 2002; Wu and Baldwin, 2010). Whereas, the insect response mechanisms involve increased biosynthesis of secondary metabolites which either directly deter insects (feeding inhibitors) such as aldehydes, lignins, tannins, terpenoids, phenolics, flavonoids, quinones, alkaloids, and glucosinolates (Mello and Silva-Filho, 2002). For such increased secondary metabolite biosynthesis, the interactions between photosynthesis, reactive oxygen species (ROS), and hormonal signaling lead to efficient resource management (Kerchev et al., 2012).

However, the knowledge of such pathways is limited in the case of RPW infected coconut plants, which is highly required to understand the key coconut response to RPW attack. Here, we have studied the leaf’s physiological and transcriptomic responses to fill this knowledge gap and provide a way forward. For this, we used the most widely cultivated coconut cultivar i.e., Hainan Tall. The information on the susceptibility or tolerance of Hainan Tall to RPW is not known. However, the earlier surveys and pilot studies by our team have indicated that if RPW infestation can be detected at an early stage, a viable RPW control strategy can be developed. In this regard, Hainan Tall plants were infected with different densities (number of insects (male and female)) on different days after infestation (DAI). First, based on leaf physiological responses, we identified the densities of the RPW that significantly affected the coconut plant. Then, we compared the transcriptomic changes in coconut leaf on 5 and 20 DAI for the selected RPW densities.

## 2 Materials and methods

### 2.2 Plant material and experimental location

Three-year-old *Cocos nucifera* cv. “Hainan tall” plants growing at the National Coconut Germplasm Nursery of Coconut Research Institute (Wenchang City, Hainan Province, China) at the Wenchang Experimental Base of Chinese Academy of Tropical Agricultural Sciences (19^○^32′15″N, 110^○^9′9″E (GCJ-02 coordinate) were used in this study. The soil at the experimental site is classified as yellow silt whose pH, organic matter, nitrogen, phosphorus, and potassium content are 6.23 ± 0.38, 30.44 ± 5.88 g/kg, 1.23 ± 0.14 g/kg, 1.57 ± 0.27 g/kg, and 9.41 ± 1.55 g/kg, respectively. Leaf samples from healthy coconut plants were sampled and used for subsequent experiments.

### 2.2 Rearing of red palm weevil, infestation density, and duration

Before the rearing of RPW, a field population dynamic monitoring of RPW was carried out for many years at the fields of Coconut Research Institute, Chinese Academy of Tropical Agricultural Science, Hainan (110^○^72′-19^○^61′). It was observed that RPW adults have three peak periods throughout the year, i.e., April to May, July to September, and November. It was also observed that RPW become active at 17:30 during the peak periods.

The RPW used in this study came from a standardized insect source raised In an artificial climate chamber (YB8-10-A) at 28°C ± 0.1°C under long day (14 h/10 h, light/dark, respectively) conditions with relative humidity of 60 ± 0.5%. The excised leaf samples were inoculated with four different RPW population densities i.e., A (5 males and seven females), B (10 males and 14 females), C (15 males and 21 females), D (20 males and 28 females) and control (CK, no RPW) on 21st April 2022 at 17:30 and after 2 days of adults mating and female oviposition (referred as infestation thereafter), coco leave samples were taken at 5 days interval, thus 5, 10, 15, 20 and 25 DAI. The triplicate leaf samples from CK and treatments were immediately frozen in liquid nitrogen and stored in the refrigerator at −80 °C until processed further.

### 2.3 Physiological index measurement

#### 2.3.1 Determination of malondialdehyde content

A total of 0.1 g fresh leaves of coco were collected and homogenized in 1 mL trichloroacetic acid. The homogenate was centrifuged at 12,000 rpm for 15 min. For every aliquot, 1 mL 20% trichloroacetic acid containing approximately 0.5% thiobarbituric acid was added and the mixture was incubated at 95°C for 30 min. The absorbance was quantified at 532 nm. Malondialdehyde (MDA) was estimated based on the previously described procedure by Heath and Packer (1968).

#### 2.3.2 Determination of enzyme activities

For extracting antioxidant enzymes, 0.5 g frozen leaves were ground in 5 mL of 50 mM cooled phosphate buffer. The homogenate was centrifuged at 1,500 rpm for 15 min at 4°C and the supernatant was used for enzymatic activity assay.

##### 2.3.2.1 Catalase and peroxidase

A total of 0.1 mL enzyme extract was thoroughly mixed with a 3 mL catalase (CAT) reaction solution dissolved in 50 mM phosphate buffer (pH 7.0) and 5.9 mM H_2_O_2_ (Wang et al., 2014). On the other hand, 0.1 mL enzyme extract was thoroughly mixed with peroxidase (POD) reaction solution dissolved in 50 mM phosphate buffer (pH 5.0), 20 mM guaiacol, and 40 mM H_2_O_2_ (Young Lee and Soo Kim, 1994). The absorbance was measured at every 20 s, and the activities of CAT and POD were computed. One unit of CAT or POD activity was defined as the absorbance per minute.

##### 2.3.2.2 Superoxide dismutase

A total of 50 μL enzyme extract was mixed with 3 mL of 50 μM nitro-blue tetrazolium, 1.3 μM riboflavin, 13 mM methionine, 75 nm ethylenediaminetetraacetic acid, and 50 mM phosphate buffer. The mixture was irradiated under fluorescent lamps at 78 μmols^-1^m^-2^ for 15 min. The absorbance was recorded at 560 nm and the activity of superoxide dismutase (SOD) was quantified following the method developed by Constantine and Ries (1977).

### 2.4 Transcriptome profiling

#### 2.4.1 Extraction of RNA and sequencing

Based on the results of the CAT, POD, and SOD activities, triplicate leaf samples from only C and D RPW population densities together with the CK sampled at 5, 10, 15, 20, and 25 DAI were used to isolate total RNAs using a Tiangen RNAprep Pure Plant Kit (Tiangen, China) following the manufacturer’s protocols. The RNA quality was assessed with NanoDrop (Thermo Scientific, Waltham, MA, United States). The cDNA of the 18 samples (2 RPW population densities × 5 DAIs × 3 biological repeats) were prepared according to the manufacturer’s protocols (NEB Next RNA Library Prep Kit). The quality and quantity of the cDNA libraries for were determined with the Agilent 2,100 bioanalyzer system (Agilent Technologies, Palo Alto. CA, United States). The cDNA libraries were sequenced by Illumina paired-end sequencing technology with a read length of 100-bp on an Illumina HiSeq 2000 instrument.

#### 2.4.2 Sequencing data analysis

Prior to data analysis, quality control checks were carried out. The raw reads were filtered by eliminating low-quality reads in the FastQC program with the default settings (http://www.bioinformatics.babraham.ac.uk/projects/fastqc/; accessed on 12th June 2022), and the clean data were then subjected to further analyses. The high-quality raw reads of each library were mapped to the coconut genome (Wang et al., 2021) using HISAT2 software with default settings (Kim et al., 2015). Raw counts of transcripts were undertaken *via* featureCounts (Liao et al., 2014). The detected transcripts were annotated to eight public databases, i.e. gene ontology (GO) (Ashburner et al., 2000), protein families (Pfam) (Bateman et al., 2004), European Molecular Biology Laboratory (TrEMBL) (Junker et al., 2000), non-redundant (Nr) (Deng et al., 2006), EuKaryotic Orthologous Group (KOG) (Tatusov et al., 2003), Kyoto Encyclopedia of Genes and Genomes (KEGG) (Kanehisa and Goto, 2000), SwissProt (Apweiler, 2001), and Plant transcription factor (version 3.0) (Jin et al., 2014).

**FIGURE 1:**
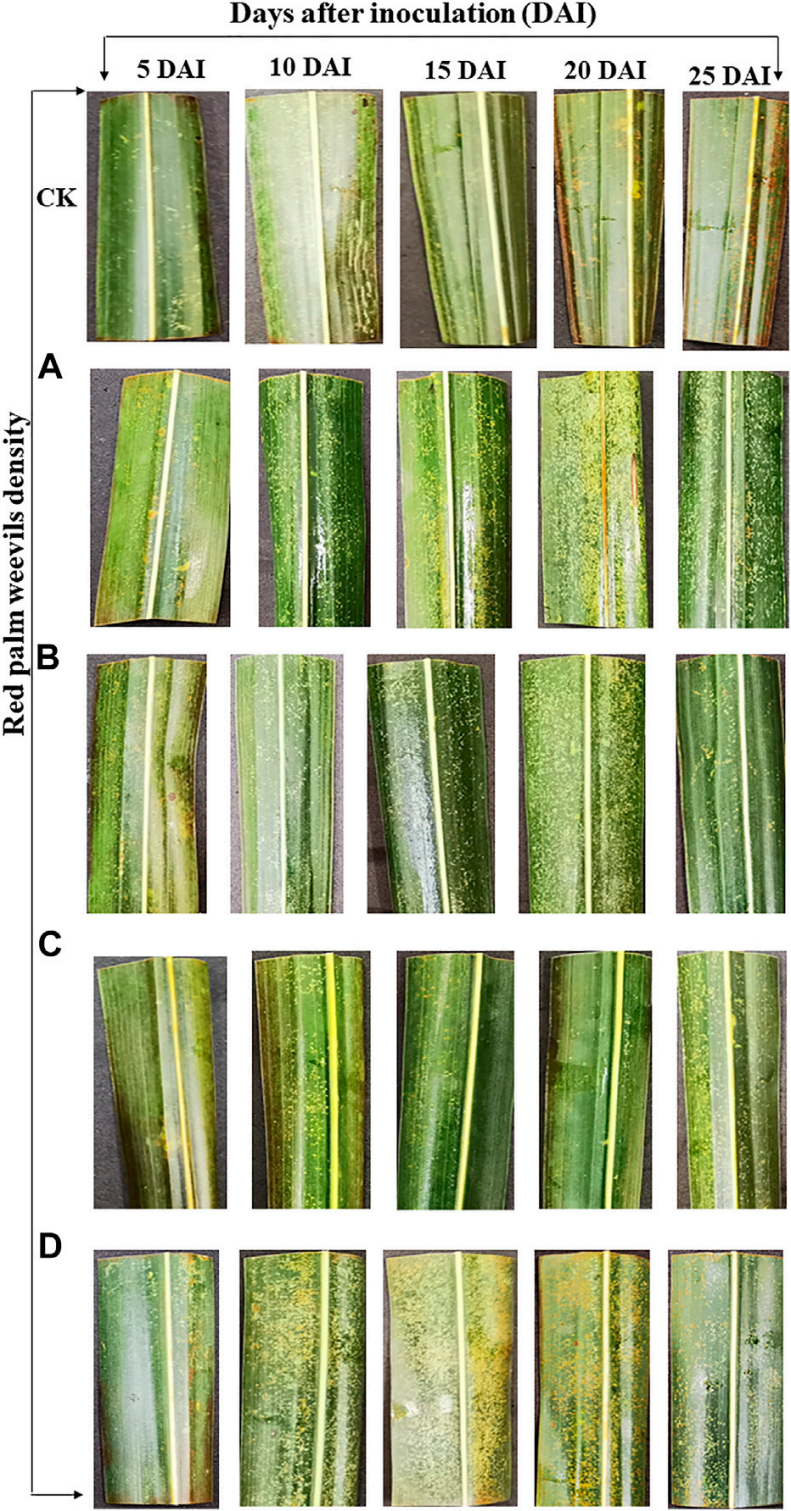
Leaf samples of *Cocos nucifera* from different red palm weevil (RPW) population densities and sampling times after infestation used for transcriptome profiling. Where, CK = no RPW; **(A)** = 5 males and seven females; **(B)** = 10 males and 14 females; **(C)** = 15 males and 21 females; **(D)** = 20 males and 28 females.

Principal component analysis (PCA) and Pearson correlation were computed using the fragments per kilobase of transcript per million fragments mapped (FPKM) values in R with the packages *corrplot* and *ggbiplot*, respectively (R Core Team, 2018). To identify the differentially expressed genes (DEGs), a stringent threshold of log2 foldchange ǀlog2FCǀ ≥1 and false discovery rate (FDR) ≤ 0.05 (Zhu et al., 2021) were adopted using *DESeq2* package in R (Love et al., 2014) (Figure 1).

### 2.5 Statistical analysis

Data collected on MDA, CAT, PODm and SOD were subjected to a two-way analysis of variance (ANOVA) and Post hoc mean separation was carried out with Duncan Multiple Range Test (DMRT) at (*p* ≤ 0.05) in GenStat, 12th edition (VSN International Ltd., UK).

### 3.2 Results *C. nucifera* leaf physiological indicators in response to red palm weevil infestation

**FIGURE 2:**
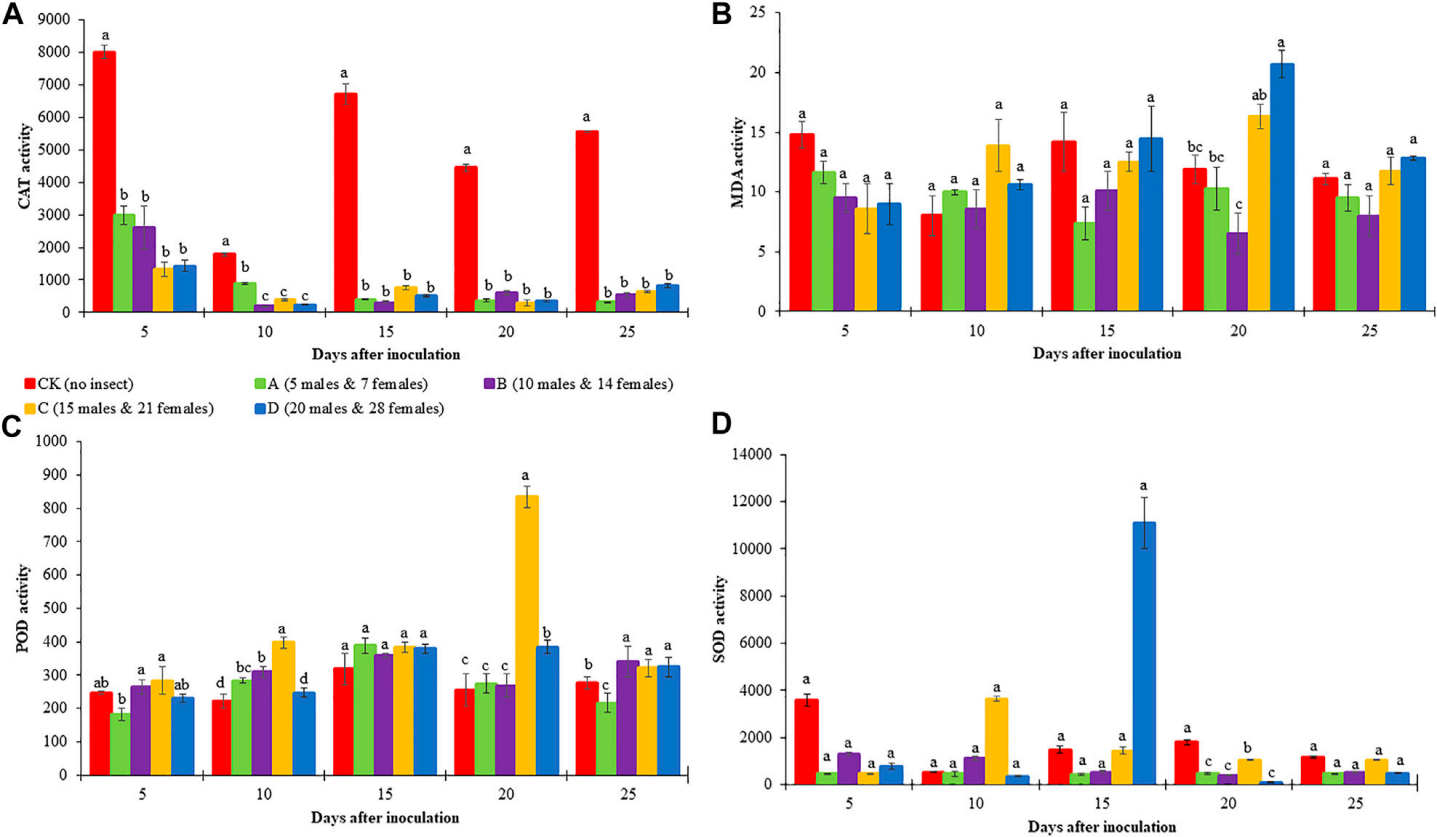
Enzyme activity in the leaf of *Cocos nucifera* infested with different densities of red palm weevil (RPW). **(A)** Catalase (CAT), **(B)** Malondialdehyde (MDA), **(C)**, Peroxidase (POD), and **(D)** Superoxide dismutase (SOD). Each result represents the average of three replicates. The error bars represent the standard error of the mean (SEM). Bars in each sampling time with the different alphabets indicate significant differences (*p* > 0.05).

Four different red palm weevil (RPW) population densities i.e., A (5 males and seven females), B (10 males and 14 females), C (15 males and 21 females), and D (20 males and 28 females), and CK (no RPW) were used to infect *C. nucifera* leaves. The leaf physiological response such as the activities of CAT, POD, SOD, and MDA content were observed in RPW infected and CK on different DAI. Coconut leaf CAT activity in CK was significantly higher than A-D RPW population densities. Generally, the CAT activity of RPW A was higher (but non-significant differences) on 5 and 10 DAI as compared to 15-25 DAI, where it decreased with an increase in RPW population density. The % decrease in CAT activity was highest in C (83.39%), B (89%), C (95%), C (93.11%), and A (94%) on 5, 10, 15, 20, and 25 DAI, respectively. These observations indicate that: 1) the increase in DAI leads towards more decrease in % CAT activity regardless of insect density, 2) overall, the highest average % decrease (average of all DAI) was observed in the case of C (86.23%) and D (87.52%) population densities but still average % decrease in A-D did not differ significantly, and 3) the effect of insect density on different DAI is different and lacks any typical trend (Figure 2A).

Coconut leaf POD activities between the different RPW population densities showed slight variations on 5, 10, 20, and 25 DAI. Generally, POD activities were lower in CK as compared to A-D with some exceptions i.e., B showed a decrease in POD activity as compared to CK on 5 and 25 DAI. Interestingly, coconut POD activity on 20 DAI for C was the highest. The % increase in POD activity was observed in C (15%), C (80%), A (22%), C (227%), and B (23%) on 5, 10, 15, 20, and 25 DAI, respectively. Overall, C had the highest % increase (69%) (average of all DAI) in POD activity followed by D, B, and A (Figure 2B). The coconut leaf SOD activities were higher in CK as compared to A-D on 5, 20, and 25 DAI. As far as SOD % increase/decrease is concerned in different insect densities as compared to CK, we found that C had a maximum % increase (89%, average of all DAI), whereas it decreased in other RPW population densities (Figure 2C).

The MDA content showed variable trends such that on five DAI, it was higher in CK as compared to A-D, whereas on 10, 20, and 25 DAI, it was lower in CK as compared to most of the RPW population densities. We observed an increase in MDA content from A-D on 15, 20, and 25 DAI with slight variations. Considering the average of all the DAI, the % MDA content increase was highest in D, which slightly differed (non-significant) from that of C, indicating that this RPW population density initiates the highest changes in MDA (Figure 2D).

From all these observations, we can understand the C and D RPW population densities affect the leaf physiological parameters on a larger scale as compared to other population densities. Furthermore, the % CAT activity decrease was minimum on five DAI and maximum on 15-25 DAI (non-significant differences), the % SOD activity increase was the least on five DAI and highest on 20 DAI, and % POD activity decrease was lowest on five DAI and highest on 20 DAI. Therefore, we explored the leaf transcriptome in response to C and D infestation on 5 and 20 DAIs.

### 3.2. Overview of transcriptome analysis of coconut leaf samples at 5 and 20 days after RPW infestation

Transcriptome sequencing (Illumina NovaSeq600) of 18 libraries was used to identify genes and their biosynthesis pathways that may be responsible for the variation in the extent of damage to coconut leaves caused by RPW infestation. The average number of raw reads and clean reads were 51,424,881 bp and 50,673,271 bp, whereas the average clean reads were 7.60 Gb per library (Supplementary Table S1). The average error rates, Q20%, and guanine-cytosine content were 0.03, 97.95, and 47.42%, respectively (Supplementary Table S1). The obtained clean reads were mapped to the *C. nucifera* reference genome for quantification (Wang et al., 2021). A total of 38,432 annotated genes’ expressions were detected and reported in terms of the FPKM values. FPKM distribution and hierarchical clustering heatmap of detected genes are shown in Supplementary Figures S1A, B. The PCA based on FPKM and Pearson correlation coefficients (PCC) (r ≥ 0.92) revealed high levels of reproducibility of samples (Supplementary Figures S2A, B). Of the 38,432 detected transcripts, 2,383 could be annotated as TFs, while 32,681 transcripts were annotated to the NR database (Supplementary Figure S2C).

### 3.3 Transcriptome profile of coconut leaves at 5 and 20 days after two RPW densities infestation

**FIGURE 3:**
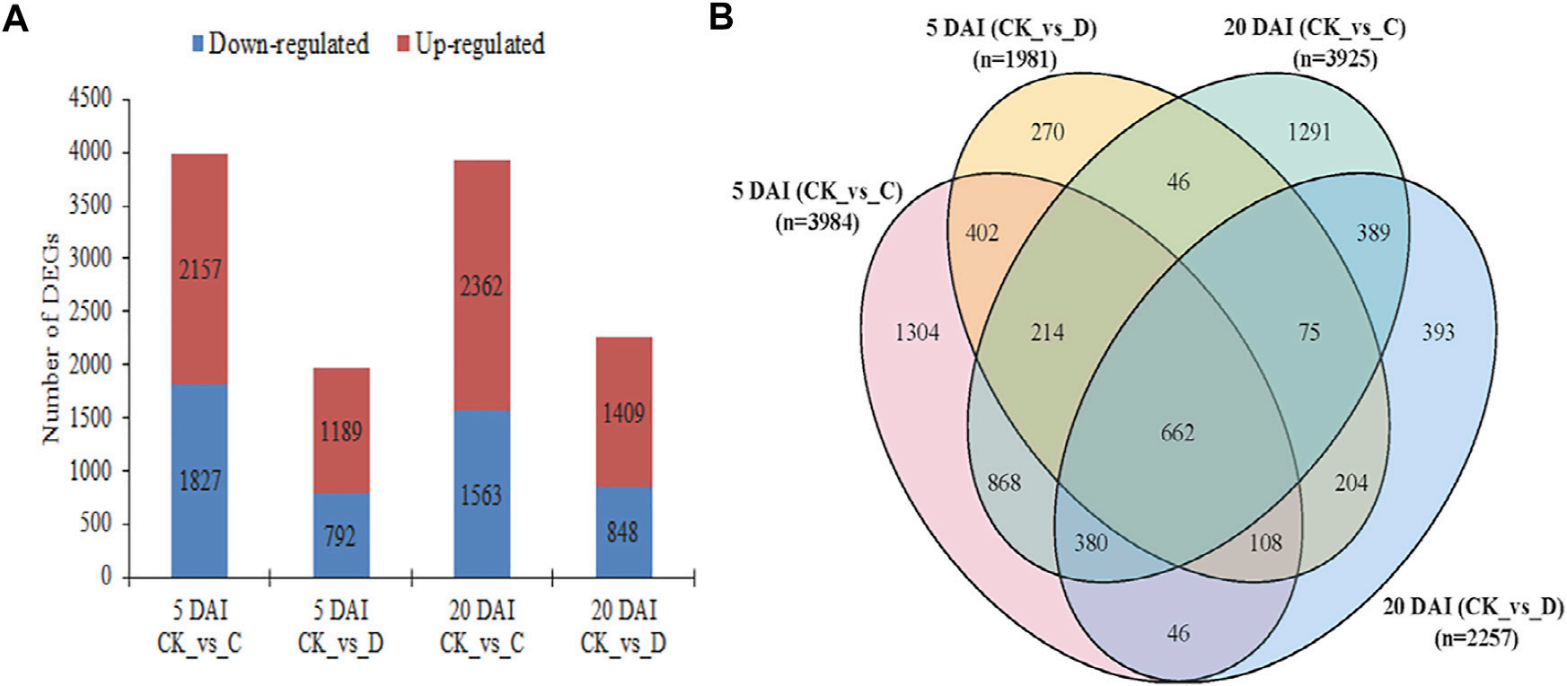
Differentially expressed genes (DEGs) in coconut leaves were detected in C (15 males and 21 females) and D (20 males and 28 females) RPW population densities as compared to the control (CK) on 5 and 20 DAI. **(A)** Bar graph of detected DEGs (both down- and upregulated). **(B)** Venn diagram of DEGs detected in the four treatment comparisons. *n* = number of DEGs detected in each pairwise group.

Based on the physiological profiles of the coconut leaves, C and D RPW population densities were found to be relatively more damaging at 5 and 20 DAI as compared to the other DAI, therefore, we further explored the key transcriptional changes in the coconut leaves at these densities and DAIs. Based on the screening criteria (log2 FC ≥ 1 and FDR ≤0.05), the total number of DEGs detected at five DAI for CK vs. C and CK vs. D were 3,984 (1,827 up and 2,157 down) and 1,981 (792up and 1,189 down), respectively. On 20 DAI, there were 3,925 (1,563 up and 2,362 down) and 2,257 (848 up and 1,409 down) DEGs in CK vs. C and CK vs. D, respectively (Figures 3A, B). It was observed that a higher number of genes were induced upon RPW attack under C and D densities relative to the CK on 20 DAI as compared to the five DAI (Figure 3A).

#### 3.3.1 Genes involved in RPW attack progression in coconut leaves

We found that 662 were commonly expressed in the four treatment comparisons (Figure 3B, Supplementary Table S2A). Among these, one pyruvate dehydrogenase phosphatase (PDP, *COCNU_15G006330*) had highest FPKM values in C population density followed by D at five DAI. However, it exhibited a decreasing expression trend at 20 DAI (C < D), whereas, its expression was not detected in CK. Moreover, three genes including dual-specificity kinase (MEK1, *COCNU_05G004480*), glutamine synthetase (GS, *COCNU_03G002950*), and 4-coumarate--CoA ligase (4CL, *COCNU_01G008610*) showed increased expressions (more than 1-fold) from 5 to 20 DAI. Additionally, two 1-deoxy-D-xylulose-5-phosphate synthases (DXS, *COCNU_scaffold008687G00001,* and *COCNU_06G013430*), four chitinases (*COCNU_scaffold006534G000080*, *COCNU_06G003500*, *COCNU_04G009610,* and *COCNU_14G001870*), two glutathione S-transferases (GST, *COCNU_01G002110* and *COCNU_07G007380*), one caffeoyl-CoA O-methyltransferase (CCoAMOT, *COCNU_08G005080*), one ferredoxin-nitrite reductase (NiR, *COCNU_scaffold000819G000030*), and one primary-amine oxidase (AOC3, *COCNU_06G014360*) exhibited increased expressions from 5 to 20 DAI. These genes and several others shown in Supplementary Table S2A are potential candidates as RPW attack progression markers.

#### 3.3.2 Potential candidate genes for effective early and late detection of RPW infestation

A total of 402 genes were expressed differentially at five DAI (Figure 3B; Supplementary Table S3B). Among these, three POD genes (*COCNU_11G004860*, *COCNU_13G003300* and *COCNU_scaffold009298G000040*), one (+)-abscisic acid 8′-hydroxylase (*COCNU_07G015720*), and two phenylalanine ammonia-lyase (PAL, *COCNU_09G010210* and *novel.12794*) were expressed highly upon RPW attack. These genes together with Jasmonate ZIM domain-containing proteins (JAZ, *COCNU_08G003270*, *COCNU_08G001960*, *COCNU_scaffold006436G000010* and *novel.10587*), and others presented in Supplementary Table S2B could be considered as potential candidate genes for early detection of RPW attack.

On the other hand, 389 genes were uniquely expressed at 20 DAI (Figure 3B, Supplementary Table S3C). Among them, one calmodulin (CaM, *COCNU_05G009180*), one aquaporin PIP (*COCNU_contig69169965G000010*), three calcium-binding protein CML (*COCNU_scaffold006994G000010, COCNU_02G012590 and COCNU_scaffold003807G000010*) and one ethylene-inducing xylanase (EIX, *COCNU_07G001140*) showed increased expression upon RPW attack. These genes and several others shown in Supplementary Table S2C could be screened further for the detection of RPW attack detection on 20 DAI.

#### 3.3.2 KEGG pathway enrichment analysis of DEGs

The DEGs in CK vs. C and CK vs. D on 5 and 20 DAI were significantly enriched in several pathways related to signaling, growth and development, and plant responses to stresses. These pathways include plant-pathogen interaction, phenylpropanoid biosynthesis, flavonoid biosynthesis, flavone and flavonol biosynthesis, isoflavonoid biosynthesis, amino sugar, and nucleotide sugar metabolism, plant hormone signal transduction, and mitogen-activated protein kinase (MAPK) signaling pathway-plant (Supplementary Table S3).

##### 3.3.2.1 Expression changes in the ROS scavenging related genes

**FIGURE 4:**
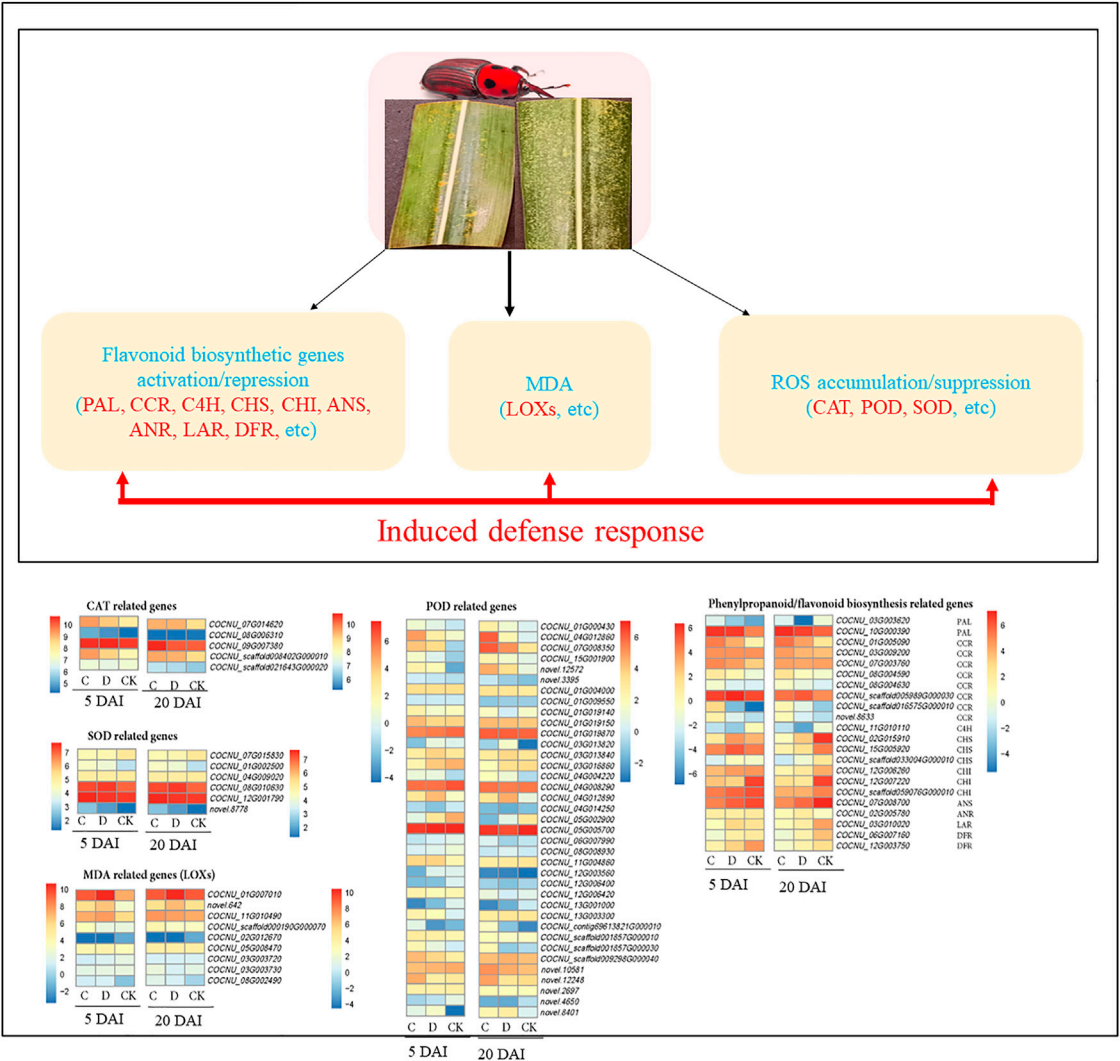
Reactive oxygen species (ROS) scavenging related gene expression changes in the two population densities of red palm weevil (RPW) as compared to CK (CK = no RPW; C = 15 males and 21 females; D = 20 males and 28 females) at 5 and 20 DAI in coconut leaves. The genes highlighted in red text were differentially expressed, POD: peroxidases; SOD: superoxide dismutase; CAT: catalase, MDA: monodehydroascorbate, and LOXs: lipoxygenase. Heatmap of log2 transformation of fragments per kilobase of exon per million fragments mapped (FPKM) values of ROS and phenylpropanoid/flavonoid biosynthesis genes with *pheatmap* package in R (Kolde and Kolde, 2018). PAL: phenylalanine ammonia-lyase [EC:4.3.1.24]; CCR: cinnamoyl-CoA reductase [EC:1.2.1.44]; C4H: trans-cinnamate 4-monooxygenase [EC:1.14.14.91]; CHS: chalcone synthase [EC:2.3.1.74]; CHI: chalcone isomerase [EC:5.5.1.6]; ANS: anthocyanidin synthase [EC:1.14.20.4]; ANR: anthocyanidin reductase [EC:1.3.1.77]; LAR: leucoanthocyanidin reductase [EC:1.17.1.3] and DFR: bifunctional dihydroflavonol 4-reductase/flavanone 4-reductase [EC:1.1.1.219 1.1.1.234]. The color gradient in the heatmaps for 5 and 20 DAI are shown on the left and right sides, respectively. The FPKM of highlighted genes and several other genes are shown in Supplementary Table S4.

To understand the basis for variation in CAT, POD, and SOD (Figure 2), we mined for genes annotated as CAT (K03781 [EC:1.11.1.6]), POD (K00430 [EC:1.11.1.7]) and SOD (K04564/K04565 [EC:1.15.1.1]). Five CAT transcripts (*COCNU_09G007380, COCNU_07G014620*, *COCNU_08G006310*, *COCNU_scaffold008402G000010,* and *COCNU_scaffold021643G000020*) expressed differentially in the CK as well as C and D densities (Figure 4; Supplementary Table S4). At 5 DAI, *COCNU_scaffold021643G000020* expression followed a trend CK > C > D, however on 20 DAI, it had the highest expression in C followed by D and CK. In addition, *COCNU_08G006310* expressed least in CK at five DAI but expressed highest in CK at 20 DAI. These two (*COCNU_scaffold021643G000020* and *OCNU_08G006310*) are likely to be responsible for the highest CAT activity observed in the CK relative to C and D densities (Figure 2A).

Conversely, the remaining three transcripts (*COCNU_09G007380, COCNU_07G014620,* and *COCNU_scaffold008402G000010*) consistently expressed highest in C followed D and least in CK in both 5 and 20 DAI, suggesting that these transcripts could be associated with the lower CAT activity as observed in the C and D densities (Figure 2A).

Thirty-seven transcripts annotated as POD were identified among the DEGs (Figure 3; Supplementary Table S4). Among them, *COCNU_01G000430*, *COCNU_04G012860*, *COCNU_07G008350*, *COCNU_05G002900, novel.12572,* and *novel.3395* exhibited the highest expressions in C followed by D and CK at both 5 and 20 DAI. These transcripts are likely responsible for the higher POD activities (Figure 2). The remaining 30 POD transcripts had varied expressions, for instance, *COCNU_13G001000* was consistently absent in the CK at both 5 and 20 DAI, whereas, *COCNU_12G003560* was absent in D and CK leaves at five DAI. These expression trends highlight their involvement in other pathways.

Six DEGs (*COCNU_01G002500*, *COCNU_04G009020*, *COCNU_07G015830*, *COCNU_08G010630*, *COCNU_12G001790* and *novel.8778*) annotated as SOD were identified from the expressed transcripts of the studied comparisons (Figure 4). From these, *COCNU_07G015830* expressed higher in CK than either C or D (both at 5 and 20 DAI), which is consistent with the SOD activity (Figure 2D). The remaining five SOD transcripts exhibited different expressions in CK, C, and D. For example, *COCNU_01G002500* and *COCNU_12G001790* had higher expression in C, followed by D, and CK, thus suggesting that these genes are upregulated upon RPW attack.

We further mined for genes that may be associated with the observed MDA content changes (Figure 2 B); involved in lipid peroxidation upon RPW attack. Nine lipoxygenases (LOXs, *COCNU_01G007010*, *novel.642*, *COCNU_11G010490*, *COCNU_scaffold000190G000070*, *COCNU_02G012670, COCNU_05G008470*, *COCNU_03G003720*, *COCNU_03G003730* and *COCNU_08G002490*) were differentially expressed. Among these, only *COCNU_02G012670*, *COCNU_03G003720,* and *COCNU_03G003730* consistently expressed highest in the CK than in both C and D population densities at five DAI (Figure 4). On the other hand, *novel.642*, *COCNU_11G010490,* and *COCNU_08G002490* were highly expressed upon RPW attack (either C or D density) as compared to the CK at 20 DAI. In addition to LOXs, 48 cytochrome P450 transcripts showed an increase in expression upon RPW attack (Supplementary Table S4).

We identified 153 DEGs involved in phenylpropanoid/flavonoid/flavone and flavonol/isoflavonoid biosynthesis in C and D population densities relative to the CK at 5 and 20 DAI (Supplementary Table S4). Two PAL genes (*COCNU_03G003620* and *COCNU_10G000390*) expressed higher in either C or D density than in CK at both 5 and 20 DAI (Figure 4). Strikingly, the former gene was absent in the CK at 20 DAI. Eight cinnamoyl-CoA reductases (CCRs, *COCNU_01G005090, COCNU_03G009200, COCNU_07G003760, COCNU_08G004590, COCNU_08G004630, COCNU_scaffold005989G000030, COCNU_scaffold016575G000010* and *novel.8633*) were highly expressed in C and D population densities than in CK at 5 as well as 20 DAI (Figure 4). CCR is documented to be involved in the defense related responses in many host-pathogen systems (Kawasaki et al., 2006; Liu et al., 2021). Trans-cinnamate 4-monooxygenase (C4H) is reported to participate in the biosynthesis of phytoalexins (Kochs et al., 1992), which helps plants to resist pathogenic infestations (Smith et al., 1971). The induced expression of C4H (*COCNU_11G010110*) upon RPW attack at both 5 and 20 DAI, indicates that *C. nucifera* attempted mitigating the attack by RPW.

Three CHS genes (*COCNU_02G015910*, *COCNU_15G005920,* and *COCNU_scaffold033004G000010*) expressed highly in D density followed by CK and C density at five DAI (Figure 4). Whereas, their expressions were higher in CK as compared to C and D densities at 20 DAI. Three chalcone isomerases (CHIs, *COCNU_12G006260, COCNU_12G007220,* and *COCNU_scaffold059076G000010*) were downregulated upon RPW attack relative to the CK at 5 and 20 DAI (Figure 4).

In this study, one anthocyanidin synthase gene (ANS, *COCNU_07G008700*) had the highest expression in CK as compared to C and D at both 5 and 20 DAI. One anthocyanidin reductase gene (ANR, *COCNU_02G005780*) followed a similar trend as of ANS at five DAI, but its expression increased in C and D as compared to CK at 20 DAI (Figure 4). Additionally, one leucoanthocyanidin reductase (LAR, *COCNU_03G010020*) expressed highest in D followed by CK and C density at five DAI. This same gene had a 2.49-fold reduction in expression upon RPW attack (C and D densities) relative to the CK on 20 DAI. Two bifunctional dihydroflavonol 4-reductase/flavanone 4-reductases (DFRs, *COCNU_06G007160,* and *COCNU_12G003750*) showed lower expressions in C and D as compared to CK (Figure 4). These expressions pinpoint that the RPW attack causes repression of anthocyanin genes*.*

Apart from those elaborated above, flavonoid 3′,5′-hydroxylase (F3′5′H), trans-cinnamate 4-monooxygenase (CYP73A5), CCoAOMT, 4CL, flavonol synthase (FLS), flavonoid 3′-monooxygenase (CYP75B1), cinnamoyl-CoA reductase (CCR), 5-O-(4-coumaroyl)-D-quinate 3′-monooxygenase (CYP98A3), coniferyl-aldehyde dehydrogenase (CALDH), shikimate O-hydroxycinnamoyltransferase (HCT), caffeic acid 3-O-methyltransferase/acetylserotonin O-methyltransferase (COMT), 2-hydroxyisoflavanone dehydratase (HID), isoflavone/4′-methoxyisoflavone 2′-hydroxylase (CYP81E7), isoflavone 7-O-glucoside-6″-O-malonyltransferase (IF7MAT), caffeoylshikimate esterase (CSE) encoded genes and several others (Supplementary Table S4) were differentially expressed. Taken together highlight that flavonoid/flavone and flavonol/isoflavonoid play a significant role in modulating *C. nucifera* response to RPW attack.

##### 3.3.2.2 Expression changes in plant pathogen interaction genes on 5 and 20 days after RPW attack

**FIGURE 5:**
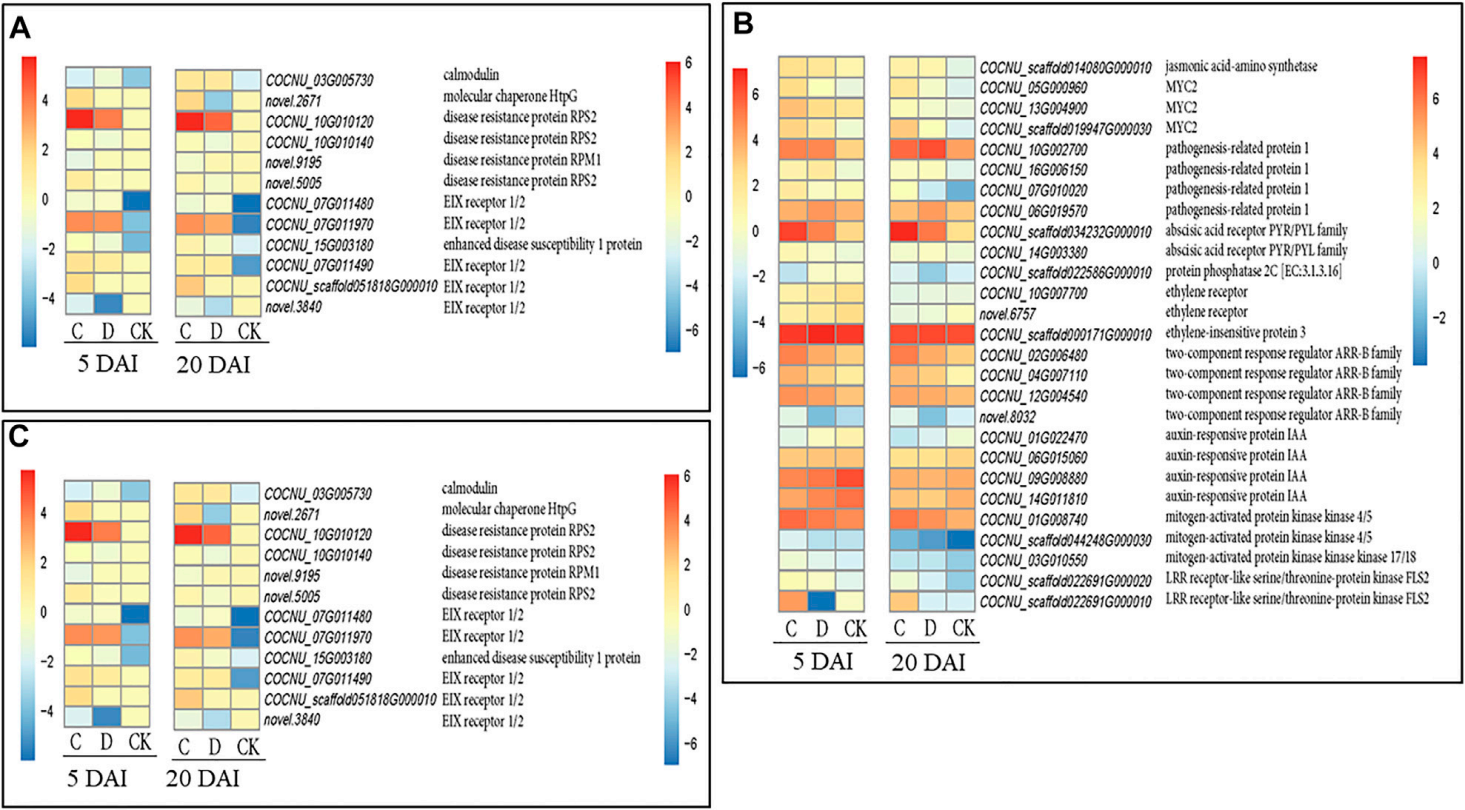
Expression profiles of potential candidate genes involved in regulating coconut leaf responses to red palm weevil (RPW) attack at 5 and 20 days after infestation (DAI) under three RPW densities (CK = no RPW; C = 15 males and 21 females; D = 20 males and 28 females). Heatmaps were produced with log2 transformation of fragments per kilobase of transcript per million fragments mapped values for each density (C, D, and CK) at 5 and 20 DAI with *pheatmap* package in R (Kolde and Kolde, 2018). **(A)**. Plant pathogen interaction genes. **(B)**. Phytohormones and MAPK cascading related genes. **(C)**. Amino sugar and nucleotide sugar metabolism related genes. The color gradient in the heatmaps for 5 and 20 DAI are shown on the left and right sides, respectively. The FPKM of highlighted genes and several other genes are shown in Supplementary Table S4.

We explored the DEGs involved in plant-pathogen interaction in C and D population densities as compared to the CK. Overall, we identified 346 DEGs in both C and D along with their CKs at 5 and 20 DAI (Supplementary Table S4). One CaM gene (*COCNU_03G005730*) expressed higher upon RPW attack (C and D) at both 5 and 20 DAI (Figure 5A). Additionally, one molecular chaperone HtpG gene (*novel.2671*) had the highest expression in C density both at 5 and 20 DAI, while this gene only expressed in D density at 20 DAI and was consistently absent in the CK.

Three disease resistance proteins RPS2 (*COCNU_10G010120, COCNU_10G010140,* and *novel.5005*), and another one disease resistance protein RPM1 (*novel.9195*) were completely absent in the CK but expressed higher in C density than D density (Figure 5). Five EIX receptor 1/2 genes (*COCNU_07G011480*, *COCNU_07G011490*, *COCNU_07G011970*, *COCNU_scaffold051818G000010* and *novel.3840*) were expressed highest in C population density than both D and CK both 5 and 20 DAI, however, these genes were completely absent/near zero expression in the CK (Figure 5A). Three EIX genes (*COCNU_07G011490, COCNU_scaffold051818G000010,* and *novel.8195*) were largely not expressed in the CK but expressed mostly in C density.

The near zero/no expression of CaM, molecular chaperone HtpG, disease resistance protein RPS2, disease resistance protein RPM1, and EIX receptor 1/2 (Figure 5) in D density could account for higher damages by RPW observed in *C. nucifera* leaf from D density than C density at both 5 and 20 DAI. Therefore, these genes together with several others encoding for DNA polymerase epsilon subunit 2, cyclic nucleotide gated channel-plant, heat shock protein 90 kDa beta, calcium-dependent protein kinase, and chitin elicitor-binding protein (Supplementary Table S4) could further be validated, and used to develop early detection of RPW infested *C. nucifera*.

##### 3.3.2.3 Changes in phytohormones and MAPK cascades in responding to *C. nucifera* to RPW attack

Plants as sessile organisms rely on a range of chemical compounds to repel enemies and attract mutualistic organisms above- and below-ground (Dicke and Baldwin, 2010). One group of such compounds comprises elaborate signaling networks regulated by phytohormones including abscisic acid (ABA), auxin, Jasmonic acid (JA), salicylic acid (SA), ethylene (ET), and cytokinins, among others. Kazan and Lyons (2014) reported that pathogens adopt innovative strategies to manipulate phytohormone-regulated defense by hijacking, evading, or disrupting hormone signaling pathways and or crosstalk. With these in mind, we explored the phytohormones and MAPK cascades related genes. Two abscisic acid receptor PYR/PYL family genes (*COCNU_10G002700* and *COCNU_16G006150*) and one protein phosphatase 2C (PP2C) gene (*COCNU_07G010020*) mostly expressed higher in C density than D density. However, the RPW attack increased the expression of these genes in C and D densities (Figure 5B; Supplementary Table S4). The relatively higher expression of ABA genes upon RPW attack (C and D densities) could contribute to the higher susceptibility of coconut. Furthermore, we detected four auxin-responsive protein IAA genes (*COCNU_01G022470*, *COCNU_06G015060*, *COCNU_09G008880,* and *COCNU_14G011810*) largely expressed in order of CK > C density > D density (Figure 5B; Supplementary Table S5). In addition to these, two auxin responsive GH3 gene family (*COCNU_01G002850* and *COCNU_05G002640*) decreased in expression from C density to D density but reduced drastically in CK. This highlights that ABA and auxin genes could either be repressors or enhancers for RPW a in *C. nucifera*.

The ET signal transduction pathway is known to be initiated by ET negatively regulating its receptors, which are a small family of five receptors (ETR1, ETR2, ERS1, ERS2, and EIN4). Two ET receptor genes (*COCNU_10G007700* and *novel.6757*) showed higher expressions in CK than C and D densities at both 5 and 20 DAI (Figure 5B). Interestingly, one ethylene-insensitive 3 (EIN3) protein (*COCNU_scaffold000171G000010*) overexpressed in leaf samples from D density followed by C density. This is not surprising as plants with overaccumulation of EIN3 are said to be compromised in pathogen-associated molecular pattern (PAMP) defenses and exhibit enhanced disease/pathogen susceptibility (Chen et al., 2009).

On the other hand, four pathogenesis-related protein 1 (PR-1) (*COCNU_06G019570, COCNU_scaffold034232G000010, COCNU_14G003380 and COCNU_scaffold022586G000010*) involved in SA signaling mostly expressed highly upon RPW attack relative to the CK both at 5 and 20 DAI (Figure 5B; Supplementary Table S4). One PR-1 (*COCNU_scaffold022586G000010*) was expressed only in C density at five DAI, but expressed both in C and D densities at 20 DAI. These proteins may account for the higher alteration of physiological indicators observed in Figure 2.

JA is well known to induce the defense enzymes such as POD (Shivaji et al., 2010; Usha Rani and Jyothsna, 2010; War et al., 2011). Density C produced highest POD activity at the five DAI (Figure 2C). Four JA-amino synthetase (JAR1) gene (*COCNU_05G000960, COCNU_13G004900, COCNU_scaffold019947G000030* and *COCNU_scaffold014080G000010*) consistently expressed higher upon RPW attack relative to the CK at both 5 and 20 DAI (Figure 5B; Supplementary Table S4).

Three cytokinin signaling related transcripts i.e., two-component response regulator ARR-B family genes (*COCNU_02G006480*, *COCNU_04G007110* and *COCNU_12G004540*) were highly expressed upon RPW attack as compared with the CK (Figure 5B). Additionally, *novel.8032* was expressed highest in the CK followed by C and D densities at five DAI, its expression was completely absent in CK at 20 DAI. Taken together, these expressions highlight the possible involvement of a crosstalk among phytohormones in regulating coconut responses to RPW attack.

Two mitogen-activated protein kinase kinase 4/5 (MAPKK4/5, *COCNU_01G008740* and *COCNU_scaffold044248G000030*) were highly expressed highest in C as compared to both D and CK both at 5 and 20 DAI (Figure 5B; Supplementary Table S4). Another MAPKK kinase 17/18 (MAPKKK17/18, *COCNU_03G010550*) was expressed in order of C density > D density > CK (Figure 5B; Supplementary Table S5).

In addition to MAPKK4/5, one LRR receptor-like serine/threonine-protein kinase FLS2 genes (*COCNU_scaffold022691G000020*) were induced largely in C density but were completely repressed in leaf samples from D density at 5 and 20 DAI, while another LRR receptor-like serine/threonine-protein kinase FLS2 (*COCNU_scaffold022691G000010*) expressed in order of C density > CK, but completely absent/near zero expression in the D density (Figure 5B; Supplementary Table S4).

The above genes suggest that C density activated a number of signaling genes involve in defense relative to D density.

##### 3.3.2.4 Expression changes in amino sugar and nucleotide sugar metabolism related genes after 5 and 20 days of RPW infestation

We mined for DEGs enriched in amino sugar and nucleotide sugar metabolism and studied their expression changes in both C and D densities of RPW relative to the CK at both 5 and 20 DAI (Figure 5C; Supplementary Table S4). Six chitinase (*COCNU_04G009630*, *COCNU_06G018650*, *COCNU_06G018680, COCNU_14G001870, COCNU_scaffold006534G000050* and *COCNU_scaffold006534G000060*) and one basic endochitinase B gene (*COCNU_scaffold006850G000050*) were mostly expressed higher in leaf samples from C density relative to either D density or CK samples.

In addition to above, one cytochrome-b5 reductase (COCNU_12G005640), one hexokinase (*COCNU_03G014710*), one fructokinase (*COCNU_01G021630*), one glucose-1-phosphate adenylyltransferase (*COCNU_16G006330*), one UDP-glucuronate decarboxylase (*novel.6745*) and one GDP-D-mannose 3′, 5′-epimerase (*COCNU_06G015260*) expressed in order of C density > D density > CK (Figure 5; Supplementary Table S4). Also, one UDP-glucose 4,6-dehydratas (*COCNU_14G002620*), three alpha-1,4-galacturonosyltransferase (*COCNU_11G011170*, *COCNU_14G009380* and *COCNU_scaffold006354G000010*) and one Ca^2+^/H^+^ antiporter (novel.4118) had the highest expression C density followed by D density and CK (Figure 5C; Supplementary Table S4).

Taken together above results, suggest that RPW attack on coconut leaves induced several transcriptional changes which could be targeted for the development of early detection tools to minimize the spread to a new area/country.

### 3.4 RPW attack causes expression changes in major transcription factor families

Eighty-six AP2/ERF genes were identified with contrasting trends of expression (Supplementary Table S5). For example, AP2/ERFs showed reduced expressions upon RPW attack; notably *COCNU_05G006140*, *COCNU_05G008070*, *COCNU_14G002720*, *COCNU_scaffold020996G000010* and *COCNU_08G004820* had lower expression in C and D as compared to CK. Whereas, two transcripts (*COCNU_contig69321237G000010* and *COCNU_01G010470*) had higher expressions upon RPW attack at both 5 and 20 DAI. These indicate that ethylene could be a potential regulator coconut leaf response to RPW attack.

A total of sixty bHLH genes identified, of which, the *COCNU_scaffold000339G000060* and *novel.7787* had higher expressions upon RPW attack both at 5 and 20 DAI (Supplementary Table S5). While two other transcripts (*COCNU_scaffold025043G000020* and *COCNU_scaffold028350G000010*) were repressed upon RPW attack. The remaining 56 transcripts had mix expression trends. Among the MYB genes, only *COCNU_08G009590*, *COCNU_06G004870,* and *COCNU_03G003930* expressions were enhanced upon RPW attack (Supplementary Table S6). While the majority of the remaining 109 MYB transcripts’ expressions were generally increased upon RPW attack. Four WRKY genes (*COCNU_02G003150, COCNU_04G001660*, *COCNU_04G012550* and *COCNU_06G001200*) were upregulated upon RPW attack as compared to CK. While remaining 63 WRKY transcripts showed varied expressions in C and D on both DAIs upon RPW attack (Supplementary Table S5).

## 4 Discussion

The increase in the area damaged by RPW is threatening the coconut industry in China (Ge et al., 2015). Continued efforts are needed for its effective control. In this regard, we attempted to understand changes in the activities of antioxidative enzymes and the global transcriptome profiles of the coconut leaves infected with RPW. Our results provide the basis for understanding the possible defense strategies in the host plant.

Firstly, our results that the RPW attack caused the reduced activity of CAT is interesting. These findings are consistent with those reported in *Pinus sylvestris* in response to wounding caused by sawfly (*Diprion pini*) oviposition (Bittner et al., 2017). The changes in expressions of CAT transcripts (*COCNU_scaffold021643G000020* and *OCNU_08G006310*) in CK are consistent with these findings (Figure 4). These results suggest that the RPW attack on different DAI causes changes in ROS generation/degradation. Which is also evident from the changes in the activities of POD and SOD in addition to CAT (particularly) (Figure 2). The observations that we did not find a specific trend between the RPW population density and DAI are not novel and have been previously reported in two coconut cultivars (PANDAN and MATAG from Malaysia) (Hasim and Yusuf, 2017). The general trend is that POD activities were lower in CK as compared to A-D is consistent with reports that plants infested with chewing insects (e.g., *Spodoptera*) exhibit increased POD activities (Singh et al., 2013). Peroxidases are pathogenesis-related proteins and have been potentially implicated in plant-insect interaction (Kehr, 2006). The increase in POD activities, particularly, on 5 and 20 DAI in C and D as compared to CK, and the consistent expressions of POD transcripts are important results that indicate their role in defense against RPW. It is also a potential indicator of infestation level. Other than these, the higher MDA content on 10, 20, and 25 DAI in RPW-infested coconut leaves as compared to CK is indicative of peroxidation of lipids. The study by (Hasim and Yusuf, 2017) also indicated somewhat similar results. The polar lipids form lipid bilayer and hence act as permeability barrier of the cells. In case of higher ROS levels, lipid peroxidation increases, which affect the normal functioning of cells. Since MDA is the final product of lipid oxidative modification (Hasim and Yusuf, 2017), the higher MDA contents on the said days and consistent expression of two LOXs (*novel.642*, *COCNU_11G010490* and *COCNU_08G002490*) indicates RPW attack can induce changes in ROS homeostasis, and may result in membrane damage (Figure 4; Supplementary Table S4). Taken together, the RPW attack initiates changes in ROS levels, which trigger differential activities of antioxidant enzymes i.e., CAT, POD, and SOD.

Survival and the performance of herbivores on plants tissues are linked with the phytoalexins i.e., (iso)flavonoids, terpenoids, and alkaloids. These secondary metabolites are directly associated with plant fitness during insect attack (Walling, 2000; Sosa et al., 2004). Thus, any expression changes in the associated genes/pathways are important to understand how coconut leaves respond to RPW attack. The observations that PAL, CAD, and C4H transcripts were highly expressed in C and D population density of RPW are important from defense point of view. PAL is the key enzyme that assists in inducing the synthesis of SA, which causes systemic resistance in many plants (Kim and Hwang, 2014). An earlier investigation showed that wounding by chewing insect (aphid) induced increased PAL expression, which lead to higher SA content; the SA content was positively correlated with the damage level (Chaman et al., 2003). Additionally, the CCR expression was also shown to be related to the SA biosynthesis, thus, a similar mechanism in coconut leaves can be present (Tronchet et al., 2010). Similarly, C4H and CAD are reported to participate in the biosynthesis of phytoalexins (Kochs et al., 1992; Tronchet et al., 2010). Thus, the expression changes in PAL, CAD, and C4H are an indication of defense activation in coconut leaves in response to RPW infestation. Further gene/pathway specific exploration can highlight the roles of PAL, CAD, and related genes in defense response against RPW. Other than these, the expression changes in a large number of transcripts associated with the phenylpropanoid and flavonoid biosynthesis pathway are consistent with a similar study of transcriptome analysis of *Phoenix canariensis* in response to RPW attack (Giovino et al., 2015). On the contrary, the lower expressions of CHI, CHS, ANS, LAR, and DFRs in C and D on both DAIs indicate that RPW attack possibly causes reduction in anthocyanin biosynthesis. An earlier study in Arabidopsis had shown the relatedness of anthocyanins with the resistance against aphid (Onkokesung et al., 2014). Nevertheless, anthocyanins play multiple roles in plants and their reduced biosynthesis may disturb the balance between photosynthesis and defense against RPW attack in coconut similar to *Pseudowintera colorata* (Menzies et al., 2016). For instance, overexpression of LAR or ANR resulted in elevation of tannins content and confers cassava resistance to two-spotted spider mite (Chen et al., 2022). Taken together, RPW attack induces expression changes in phenylpropanoid, flavonoid, and anthocyanin biosynthesis genes. Furthermore, the expression changes in higher number of genes in C and D RPW population densities on 20 DAI as compared to five DAI indicate that coconut leaves undergo higher changes in flavonoid and associated pathways when more days have passed after the infestation. In addition, the numerous potential candidate genes could be useful to breeding for resistant coco cultivars.

The higher expression of genes such as CaM, CDPK, HtpG, RPS2, RPM1, and EIX receptor 1/2 in response to both C and D population densities of RPW on 5 and 20 DAI indicate that coconut responds by activating defense mechanisms against RPW (Figure 5). Ca^2+^ signaling is crucial for plant-herbivore interaction (Parmagnani and Maffei, 2022). The higher expression of CDPK indicate that the RPW attack triggered changes in Ca^2+^ are being conveyed *via* enzymatic reactions (DeFalco et al., 2010) as well as by non-catalytic sensor relay proteins i.e., CaMs (Qiu et al., 2012). These changes are consistent with the earlier suggested roles of CaM in regulating wound-induced signaling in plants (Qiu et al., 2012). The upregulation of RPS2 and RPM1 proteins as well as HSP90 in RPW infested coconut leaves indicate that RPW have established a sustained interaction with host plant. The RPS2 and RPM1 proteins are part of plant-pathogen interaction pathway and can positively trigger increased expression of HSP90, which induce hypersensitive response (HR) (https://www.genome.jp/pathway/ko04626 (Arakawa et al., 2005)). Although HR is induced by fungi, oomycetes, bacteria, and viruses, but its induction by insects through unknown mechanisms is also reported in *Salix viminalis* against *Dasineura marginematorquens* (Höglund et al., 2005) and in wheat against Hessian fly (Aljbory et al., 2020). Thus, the transcriptome analysis of coconut leaves indicates that RPW induced changes in Ca^2+^ sensing and signaling, and HR.

Since signal transduction pathways mediated by phytohormones are one of the major defense strategies in plants against abiotic stresses, therefore, our results that multiple genes associated with plant-hormone signal transduction and MAPK-signaling (plant) are important (War et al., 2012). The changes in the expression of PYR/PYLs and PP2C indicate ABA reception and signaling in coconut leaves in response to infestation by both C and D population densities on 5 and 20 DAI (Figure 5B; Supplementary Table S4). These observations are consistent with earlier reports that ABA signaling is primary driver of oil palm against RPW attack (Harith-Fadzilah et al., 2021). However, our results also imply that other phytohormones including auxin, ethylene, SA, JA, and cytokinins are involved in coconut’s leaf responses to RPW infestation (Kunkel and Brooks, 2002). The expression changes in ET receptor genes and EIN3 indicate that ethylene signaling is involved in coconuts’ responses against RPW infestation. Since it is known that ethylene and JA work together in *Alnus glutinosa* L. (Horiuchi et al., 2003) against invading insect by enhancing terpenes and volatiles, therefore, an interplay of these hormones might also be functional in coconut leaves under RPW attack. A detailed understanding of the role of ethylene in coconut against RPW infestation is needed by exploring expressions of the above-mentioned genes. Whereas the higher expressions of the PR1 transcripts in C and D on 5 and 20 DAIs may be a result of ethylene signaling since PR1 RNA accumulation in tomato was dependent on ethylene signals (Ciardi et al., 2001). However, the changes in PR1 expressions could also be due to SA signaling as reported in Arabidopsis infested with whitefly (Zarate et al., 2007). Apart from these signaling related gene expressions, the upregulation of both MAPKKK4/5 and MAPKKK17/18 transcripts is also an important preliminary data and indicates that RPW infestation can activate these genes in coconut leaves (Figure 5B; Supplementary Table S5). Since MAPK signaling, together with JA, SA, and ethylene signaling, is required for transcriptional activation of herbivore defense-related genes and metabolites (Hettenhausen et al., 2015), therefore, coconut’s defense can be improved by manipulating these genes.

Insect attack alters carbon allocation patterns in plants in such a way that it triggers carbon remobilization from damaged to undamaged tissues, mostly towards the leaves to support defense (Schwachtje et al., 2006; Gómez et al., 2012). Increased sugar biosynthesis is essential to fuel the energy requirements for plant defense, scavenge ROS, initiate signaling cascades for activation/deactivation of defense genes, and change the hormone levels (Trouvelot et al., 2014). Our results that large numbers of transcripts enriched in amino sugar and nucleotide sugar metabolism pathways were increasingly expressed in coconut leaves after RPW infestation are relevant to the above-mentioned reports (Figure 5C; Supplementary Table S4). These expression changes indicate that RPW infestation initiated sugar metabolism both on 5 and 20 DAI in response to an attack by different RPW population densities. The general trend that on expressions on 20 DAI were higher than that of on five DAI indicates that higher sugars are being synthesize when a greater number of days have passed after infestation. These results should be further validated on more coconut varieties and after confirmation can be adapted as markers for RPW infestation (Ray et al., 2016).

The induction of a large number of TF families indicate that TFs are an essential part of the coconut’s responses to RPW infestation. Expression changes in a large number of AP2/ERF transcripts signify that ethylene signaling is an important part of the coconut leaf’s responses towards RPW infestation. Earlier studies have shown that AP2/ERFs were induced after *Spodoptera exigua* and *Pieris rapae* L. infestation (Rehrig et al., 2014). Tomato plants overexpressing AP2/ERF TF JRE4 showed increased biosynthesis of steroidal glycoalkaloids, which increased plant defense against *Spodoptera litura* larvae infestation (Nakayasu et al., 2018). On the other hand, the expression changes in bHLH, MYB, and WRKY TFs (Supplementary Table S5) present novel candidates for improving RPW resistance in coconut. Their upregulation in coconut leaves infested with C and D population densities of RPW on different DAI present them as defense related TFs. These expression changes are consistent with earlier reports on the roles of bHLH in rice against brown planthopper (Wang et al., 2019), MYB in Arabidopsis against *Helicoverpa armigora* (Shen et al., 2018), and WRKYs in rice against *Chilo suppressalis* (Li et al., 2015)*.* Taken together, our findings indicate that RPW infestation can induce a large number of TFs belonging to diverse families.

## 5 Conclusion

RPW infestation on coconut can trigger changes in ROS levels and antioxidative enzyme activities. Generally, the higher RPW population densities i.e., C and D, were more damaging to coconut leaves as compared to CK. With the increase in the number of days after infestation, the expressions of a relatively higher number of genes are induced. The pathways which are highly regulated in response to RPW attack include amino sugar and nucleotide sugar biosynthesis, flavonoid biosynthesis, plant-pathogen interaction, plant-hormone signaling, MAPK-signaling, and ROS scavenging. We present a large number of candidate genes that can be used to detect early (5 DAI) or late (20 DAI) RPW infestation in coconut. We also report a preliminary understanding of the coconut plant’s transcriptomic responses to RPW attack.

## Data Availability

The original contributions presented in the study are publicly available. This data can be found here: https://www.ncbi.nlm.nih.gov/bioproject/900006.
