# Supplementary material for: Transcriptome sequencing of Cocos nucifera leaves in response to Rhynchophorus ferrugineus infestation
Source: Front Genet. 2023 Feb 7;14:1115392. doi: 10.3389/fgene.2023.1115392 (PMC9942928; doi:10.3389/fgene.2023.1115392)
Supplement: Supplementary file 4 [file Table2.DOCX]

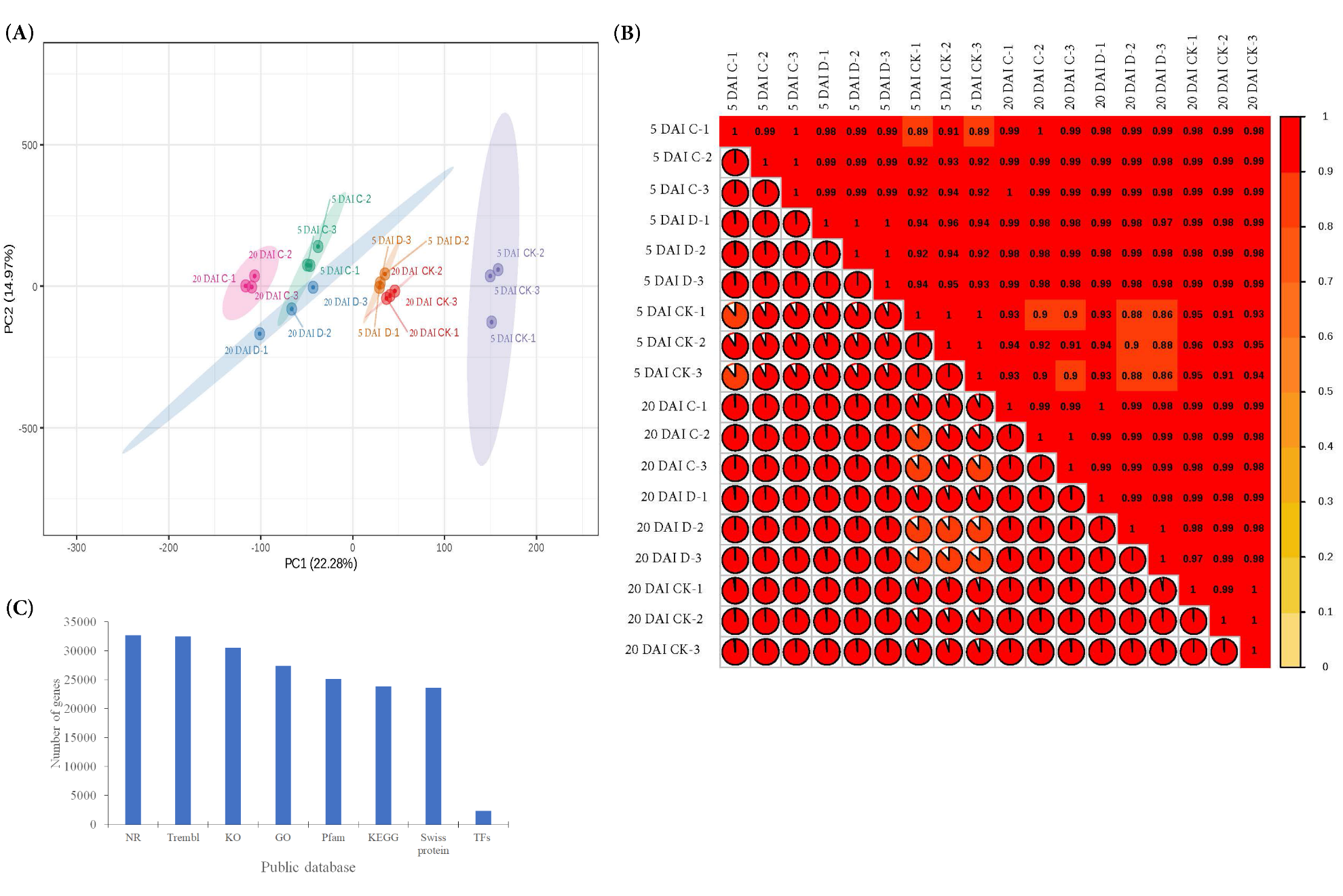


**Supplementary Figure 2:** Analysis of detected genes among the nine samples (3 red palm weevil (RPW) densities × 2 sampling times × 3 biological replicates). **A**. Principal component analysis based fragments per kilobase of exon per million fragments mapped (FPKM). **B**. Pearson correlation coefficient plot among detected genes based FPKM. **C.** Functional annotation of detected genes to public databases. RPW density (CK=no RPW; C=15 males and 21 females; D=20 males and 28 females). Sampling times consisted of 5 and 20 days after inoculation (DAI) with RPW.
